# Association Between Thyroid Dysfunction and Incidence of Atrial Fibrillation in Patients With Hypertrophic Obstructive Cardiomyopathy

**DOI:** 10.3389/fendo.2022.875003

**Published:** 2022-07-04

**Authors:** Xiangbin Meng, Xu-Liang Wang, Zhi-yuan Zhang, Kuo Zhang, Jun Gao, Ji-lin Zheng, Jing-Jia Wang, Yu-peng Liu, Jie Yang, Chen Li, Yi-Tian Zheng, Chunli Shao, Wen-Yao Wang, Yi-Da Tang

**Affiliations:** ^1^ Department of Cardiology and Institute of Vascular Medicine, Peking University Third Hospital, Key Laboratory of Molecular Cardiovascular Science, Ministry of Education, Beijing, China; ^2^ Department of Cardiology, State Key Laboratory of Cardiovascular Disease, Fuwai Hospital, National Center for Cardiovascular Diseases, Graduate School of Peking Union Medical College, Chinese Academy of Medical Sciences and Peking Union Medical College, Beijing, China; ^3^ Department of Breast Surgery, Henan Provincial People's Hospital, Zhengzhou University People's Hospital, Zhengzhou, China

**Keywords:** thyroid dysfunction, hypertrophic obstructive cardiomyopathy, atrial fibrillation, TSH, risk factors

## Abstract

**Objective:**

To explore the correlation between the incidence of atrial fibrillation (AF) and thyroid dysfunction in patients with hypertrophic obstructive cardiomyopathy (HOCM).

**Methods:**

Thyroid function testing in 755 consecutive patients with HOCM were examined at the National Center for Cardiovascular Diseases (China) from October 2009 to December 2013. Patients were divided into four groups according to the TSH levels: TSH<0.55 mIU/L(n=37)、0.55~2.49 mIU/L (n=490)、2.50~9.9 mIU/L (n=211) and >10.00mIU/L(n=17).

**Results:**

A total of 107 patients were diagnosed with AF (14%).(1) Compared to HOCM patients without AF,HOCM patients with AF have older age (P<0.001), higher NT-proBNP (P=0.002), higher Cr (P=0.005), larger left atrial diameter(P=0.001), lower FT3 (P=0.046), higher FT4 (P=0.004).(2) In the four groups according to the TSH levels: TSH<0.55 mIU/L, 0.55~2.49mIU/L, 2.50~9.9mIU/L and ≥10.00mIU/L, the incidence of AF was 27.02%(10/37),10.20%(50/490), 19.43%(41/211), and 35.29%(6/17), respectively. Both high and low TSH levels were associated with an increased incidence of AF. After adjusting for the common risk factor (age, NT-proBNP, and so on), stepwise multiple logistic regression analysis revealed that TSH levels were significantly related to AF incidence.Compared to patients with TSH 0.55~2.49 mlU/L, the adjusted odds ratio of AF for TSH<0.55, 2.50~9.99, ≥10.00 mIU/L were 1.481 (95% CI 0.485~4.518,P=0.490), 1.977 (95%CI 1.115~3.506, p=0.02), 4.301 (95%CI 1.059~17.476, P=0.041), respectively.

**Conclusion:**

Our results suggested that thyroid dysfunction was associated with an increased risk of AF in patients with HOCM.

## Introduction

More and more studies have shown that thyroid dysfunction is a significant risk factor for the progression of cardiovascular disease (1, 2). Our previous research and other clinical evidence suggest (3, 4), The level of thyroid hormone is related to the deterioration of cardiac function and the occurrence of arrhythmia. Hypertrophic cardiomyopathy (HCM) is a kind of cardiomyopathy characterized by asymmetric myocardial hypertrophy, which usually occurs at the base of the interventricular septum and the lateral wall of the left ventricle (5). HCM has a variety of clinical manifestations, can occur in all age groups and has a familial genetic tendency, which is a common cause of sudden cardiac death. Heart failure and electrophysiological disorders may occur in the later stage of the disease (6–8). Atrial fibrillation is the most common persistent arrhythmia in patients with HCM. It has been found that 2% to 3.8% of HCM patients are newly diagnosed with atrial fibrillation each year, which increases the risk of heart failure, stroke/embolism, and death, especially in patients with hypertrophic obstructive cardiomyopathy with left ventricular outflow tract obstruction (9). Left ventricular filling in patients with HCM mainly depends on atrial contraction. Atrial fibrillation will shorten the left ventricular filling time and damage the left ventricular diastolic function, resulting in frequent hospitalization and declining quality of life. Atrial fibrillation is one of the risk factors for the poor prognosis of HCM. Therefore, the clinical management of hypertrophic cardiomyopathy complicated with atrial fibrillation is a very important topic (10).

However, only a few clinical studies have shown that thyroid dysfunction is related to left ventricular diastolic dysfunction in patients with HCM (11). There is no study on the role of thyroid function in predicting atrial fibrillation in patients with HCM. This study was based on 756 patients with hypertrophic obstructive cardiomyopathy to investigate the relationship between thyroid function and atrial fibrillation in patients with hypertrophic obstructive cardiomyopathy.

## Methods

### Ethics Statement

The study followed the ethical guidelines of the declaration of Helsinki and China’s regulations and policies on good clinical practice. The Ethics Committee approved it of the Fuwai Hospital. Before the study, we got written informed consent from all participants.

### Study Patients

All patients in this study were evaluated at the Fuwai Hospital (National Center of Cardiovascular Diseases, China). Between October 1, 2009, and December 31, 2013, a total of 824 patients (age≥16 years) were diagnosed with HOCM. Among those participants, 755 subjects, with complete information on thyroid function, clinical information, and medical history, in the absence of any other cardiac or systemic disease capable of producing the magnitude of hypertrophy evident, such as uncontrolled hypertension (home blood pressure monitoring ≥140/90 mmHg), congenital heart disease, cardiac valve disease, and amyloidosis, were selected. The diagnosis of HOCM was based on (12, 13): (1) Echocardiography showed asymmetric interventricular septal thickening > 13 mm, and interventricular septal (IVS)/left posterior ventricular wall (LVPW) > 1.3 or left ventricular apical or free wall localized thickening > 15 mm. (2) tissue Doppler echocardiography, and MRI showed hypertrophy of apical and near apical IVS, dense myocardium or disordered interstitial arrangement. (3) echocardiography showed that the pressure difference of the left ventricular outflow tract was ≥ 30mmHg.

This study passed the review of the Medical Ethics Committee of Fuwai Hospital of the Chinese Academy of Medical Sciences, and all the subjects signed informed consent forms.

### Collect Clinical Data

The demographic data (age, sex, weight, height), lifestyle (smoking history, drinking history), basic heart disease history, concomitant diseases (hypertension, diabetes, stroke, ventricular arrhythmia), (NYHA) cardiac function classification of New York Cardiology Association, electrocardiogram (admission ECG, postoperative ECG) and echocardiography were collected through the electronic medical record system of Fuwai Hospital. Thyroid hormone determination equipment adopts ADVIA immune detection system produced by Siemens. The levels of serum thyrotropin (TSH), free triiodothyronine (FT3), free thyroid hormone (FT4), total triiodothyronine (TT3), and total thyroid hormone (TT4) were detected by the Immunochemical luminescence method. The kit used was a Siemens kit. The normal reference values of thyroid hormones are as follows: TSH:0.55~4.78mIU/L, FT3:2.76~6.30 pmol/L.FT4:1.23~2.90pmoL/L,TT3:1.00~2.94nmol/L.TT4:55.34~160.86nmol/L.All subjects received a full set of laboratory tests simultaneously, including blood lipids, liver and kidney function, blood glucose, NT-proBNP, and so on.

### Definition of Thyroid Function and Diagnosis of Atrial Fibrillation

TSH, FT3, FT4, TT3, and TT4 are all defined as normal thyroid function in the normal reference range. Hypothyroidism (hypothyroidism) is elevated TSH levels, with FT3, FT4, TT3, and TT4 levels within or below the normal reference range. In contrast, hyperthyroidism (hyperthyroidism) decreases TSH levels, with FT3, FT4, TT3, and TT4 levels in or above the normal reference range. In addition to the routine grouping methods (clinical and subclinical hyperthyroidism, clinical and subclinical hypothyroidism, and normal thyroid function), some researchers carry out grouping analysis according to TSH level, based on these previous literature reports and expert consensus (14). In this study, TSH, the most sensitive indicator of thyroid function, was divided into three groups: TSH < 0.55, 0.55 ± 2.49, 2.50 ± 9.99, and > 10.00mIU/L. The diagnosis of paroxysmal atrial fibrillation and persistent atrial fibrillation was based on the 2010 European ESC guidelines for diagnosing and treating atrial fibrillation (15).

### Data Analysis

Statistical analysis was assessed with SPSS 21.0 statistical package for Windows. All continuous variables are presented as means ± SD, and analysis of variance was used to compare means across multiple groups. The relationships between parametric variables were assessed by multiple linear regression analysis. Initial differences in baseline characteristics between achieved treatment groups were sought in a bivariable investigation using χ2 tests, Fisher exact tests, and Student t-tests. Univariate and multivariate logistic regression analysis was used to explore the relationship between thyroid function and atrial fibrillation in patients with hypertrophic cardiomyopathy.

## Results

### Study Population and Baseline Clinical Characteristics

Seven hundred fifty-six people were included in this study, including 456 males and 300 females. **Table 1** summarizes all the selected subjects’ general clinical data, thyroid hormone levels, and echocardiography. The patients were divided into two groups according to whether they had atrial fibrillation or not: hypertrophic obstructive cardiomyopathy with atrial fibrillation (n=107) and hypertrophic obstructive cardiomyopathy without atrial fibrillation (n = 649). The incidence of atrial fibrillation in this study population was 14%. Patients with hypertrophic obstructive cardiomyopathy with atrial fibrillation were older than patients with simple hypertrophic obstructive cardiomyopathy (p<0.001). The levels of serum creatinine, NT-proBNP, and FT4 were higher (p<0.05), but the level of FT3 was lower (p=0.046) (**Table 1**).

**Table 1:** Clinical baseline characteristics of patients with hypertrophic obstructive cardiomyopathy with or without atrial fibrillation.

	Hypertrophic obstructive cardiomyopathy with atrial fibrillation (n= 107)	Hypertrophic obstructive cardiomyopathy without atrial fibrillation (n=648)	P-value
Age (years)	56.99±11.73	50.18±12.81	<0.001
Female (n, %)	43 (40.19)	257 (39.60)	0.908
BMI (kg/m2)	26.00±5.10	25.73±5.94	0.688
Hypertension disease (n, %)	41 (38.32)	221 (34.05)	0.403
Diabetes history (n, %)	6 (5.61)	42 (6.47)	0.729
History of hyperlipidemia (n, %)	32 (29.90)	191 (29.43)	0.935
A clear family history of HCM (n, %)	9 (8.41)	40 (6.16)	0.396
Drinking history (n, %)	33 (30.84)	186 (28.66)	0.680
Smoking history (n, %)	48 (44.86)	294 (45.30)	0.879
Systolic blood pressure (mmHg)	122.10±18.00	120.98±5.94	0.563
Diastolic pressure (mmHg)	74.59±11.47	73.90±11.43	0.560
Heart rate (b.p.m.)	71.50±13.13	71.69±27.59	0.944
LDL-C (mmol/L,x±s)	2.48±0.89	2.35±0.93	0.186
HDL-C (mmol/L,x±s)	0.96±0.30	0.97±0.33	0.809
Triglyceride (mmol/L,x±s)	1.59±0.88	1.68±0.98	0.354
Total cholesterol (mmol/L,x±s)	4.12±1.11	4.02±1.11	0.428
NT-proBNP (fmol/mL)	2476.08±1808.93	1814.85±1712.95	0.002
Serum creatinine (µmol/L)	82.77±23.73	76.56±20.05	0.005
TSH (mIU/L)	3.02±3.82	2.45±4.10	0.180
FT4 (ng/dL)	1.26±0.26	1.18±0.23	0.002
FT3 (pg/mL)	2.88±0.59	2.99±0.54	0.046
TT4 (ng/mL)	8.00±1.93	7.81±1.79	0.322
TT3 (ug/dL)	1.02±0.33	1.08±0.29	0.092
Echocardiography			
RV end-diastolic diameter (mm)	21.74±5.77	20.28±4.38	0.003
LA diameter (mm)	44.82±8.04	39.25±13.28	0.001
Interventricular septal thickness (mm)	19.79±4.52	20.27±5.68	0.415
LV end-diastolic diameter (mm)	42.65±6.21	42.49±6.02	0.801
LV posterior wall thickness (mm)	11.86±2.77	11.94±2.90	0.780
LV ejection fraction (%)	67.07±8.52	68.14±8.91	0.248
LV outflow tract gradient, at rest (mmHg)	63.42±32.70	74.56±33.42	0.002

The data in the table is expressed in the form of "mean ±SD" or "n (%)". BMI, body mass index; NT-proBNP, amino terminal pro-brain natriuretic peptide; TSH, thyrotropin; FT3, free triiodothyronine; FT4, free thyroxine; TT3, serum total triiodothyronine; TT4, serum total thyroxine.

### Baseline Data and Indicators of Patients Grouped by Different TSH Levels

According to the plasma TSH level, the patients were divided into four groups: TSH < 0.55,0.55 ~ 2.49,2.50 ~ 9.99and ≥ 10.00 mIU/L groups. There were significant differences in sex, smoking history, TC, LDL-C, TSH, FT3, FF4, left ventricular end-diastolic diameter, LVEF, and the incidence of atrial fibrillation among different TSH levels groups. The incidence of atrial fibrillation in the TSH (0.55~2.49mlU/L) group was the lowest (10.20%), while TSH(> 10.00 mIU/L) group was the highest (35.29%). In TSH (< 0.55 mlU/L) group and TSH(2.50 ~ 9.99 mIU/L) group, the incidence of atrial fibrillation was 27.02% and 19.43%, respectively. There was a significant difference between those four groups (P < 0.001). In addition, compared with the TSH (0.55 ~ 2.49 mIU/L) group (normal control group), the average level of total cholesterol and LDL-C in the abnormal TSH group was higher (p<0.05), but there was no significant difference in NT-proBNP, creatine kinase isoenzyme (CK-MB), uric acid, LAEDD and LVEDD (P > 0.05) (**Table 2**).

**Table 2:** General clinical data of patients with different TSH levels.

	TSH level (mlU/L)				P-value
	<0.55 (n=37)	0.55~2.49 (n=490)	2.50~9.99 (n=211)	≥10.00 (n=17)	
Age (years)	53.79±15.78	50.95±12.26	50.86±13.82	53.31±10.26	0.522
Female (n, %)	20 (54.05)^a^	162 (33.06)	100 (47.39)^a^	7 (41.18)	<0.001
BMI (kg/m2)	25.15±4.44	26.16±6.68	24.94±3.64^a^	26.38±3.19	0.099
Hypertension disease (n, %)	14 (37.84)	173 (35.31)	70 (33.18)	4 (23.53)	0.699
Diabetes history (n, %)	1 (2.70)	32 (6.53)	13 (6.16)	2 (11.76)	0.637
History of hyperlipidemia (n, %)	9 (24.32)	142 (28.98)	68 (32.23)	4 (23.53)	0.672
A clear family history of HCM (n, %)	2 (5.40)	32 (6.53)	13 (6.16)	2 (11.76)	0.836
Drinking history (n, %)	11 (29.73)	150 (30.61)	55 (26.07)	3 (17.65)	0.450
Smoking history (n, %)	18 (48.65)	249 (50.82)	68 (13.88)^a^	7 (41.18)	<0.001
Systolic blood pressure (mmHg)	122.03±19.83	121.43±18.38	120.70±18.51	117.24±17.94	0.803
Diastolic pressure (mmHg)	72.58±10.36	74.17±11.37	73.70±11.65	75.29±13.40	0.797
Heart rate (b.p.m.)	71.97±13.47	72.24±31.11	69.92±10.06	75.65±22.61	0.667
LDL-C (mmol/L,x±s)	2.09±0.76	2.38±0.92	2.33±0.92	3.10±1.20	0.002
HDL-C (mmol/L,x±s)	0.93±0.32	0.97±0.35	0.97±0.29	1.09±0.33	0.415
Triglyceride (mmol/L,x±s)	1.83±1.41	1.66±0.95	1.66±0.95	1.65±0.43	0.792
Total cholesterol (mmol/L,x±s)	3.80±0.86	4.04±1.10	3.99±1.11	4.84±1.38^a^	0.012
NT-proBNP (fmol/mL)	2265.36±1910.45	1802.25±1625.36	2008.33±1841.96	2326.71±2364.00	0.264
Serum creatinine (µmol/L)	78.34±28.65	77.47±18.88	75.92±20.54	83.18±19.56	0.466
TSH (mIU/L)	0.29±0.19^a^	1.45±0.51	3.73±1.15^a^	23.56±14.68^a^	<0.001
FT4 (ng/dL)	1.38±0.58^a^	1.20±0.18	1.16±0.20^a^	0.96±0.26^a^	<0.001
FT3 (pg/mL)	3.25±1.77^a^	3.00±0.38	2.91±0.40^a^	2.61±0.45^a^	<0.001
TT4 (ng/mL)	8.22±2.91	7.93±1.71	7.70±1.54	6.32±2.95^a^	0.001
TT3 (ug/dL)	1.12±0.68	1.07±0.26	1.05±0.26	1.05±0.28	0.566
Echocardiography					
RV end-diastolic diameter (mm)	21.00±4.83	20.61±4.74	20.07±4.42	20.94±3.42	0.457
LA diameter (mm) (mm)	38.69±9.07	39.79±6.66	40.67±21.63	41.76±5.79	0.706
Interventricular septal thickness (mm)	20.54±7.36	20.17±5.29	20.16±5.76	20.88±5.76	0.934
LV end-diastolic diameter (mm)	45.32±7.20	42.87±5.92	41.13±5.55^a^	41.88±7.01^a^	<0.001
LV posterior wall thickness (mm)	11.60±2.88	11.96±2.83	11.93±2.91	11.58±3.63	0.864
LV ejection fraction (%)	62.99±14.24^a^	68.36±8.54	67.97±7.93	70.69±5.28	0.002
LV outflow tract gradient, at rest (mmHg)	71.37±42.35	72.886±33.62	73.94±32.22	71.70±27.65	0.968
AF (n, %)	10 (27.02%)^a^	50 (10.20%)	41 (19.43%)^a^	6 (35.29%)^a^	<0.001

a is compared with TSH 0.55~2.49mIU/L group (normal control group).

### Univariate Logistic Regression Analysis of Thyroid Hormone Level and Atrial Fibrillation in Patients With Hypertrophic Obstructive Cardiomyopathy

Age: (OR: 1.045, 95%CI: 1.027~1.063,p<0.001), NT-proBNP(Per 100 fmol/mL):(OR: 1.017,95%CI:1.006~1.029,p=0.003),serum-creatinine:(OR:1.013, 95%CI:1.004~1.022,p=0.006),FT3:(OR:0.616,95%CI:0.392~0.968,p=0.035),FT4:(OR: 3.336, 95%CI:1.483~7.503,p=0.004) (**Table 3**).

**Table 3:** Univariate logistic regression analysis of atrial fibrillation in patients with HOCM.

	OR	95%CI	P-value
Female	1.025	0.675-1.555	0.908
Age (years)	1.045	1.027-1.063	<0.001
NT-proBNP (per 100 fmol/mL)	1.017	1.006-1.029	0.003
Serum creatinine (µmol/L)	1.013	1.004-1.022	0.006
FT3 (pg/mL)	0.616	0.392-0.968	0.035
FT4 (ng/dL)	3.336	1.483-7.503	0.004

### Multivariate Logistic Regression Analysis of TSH Level and the Risk of Atrial Fibrillation

**Figure 1:**
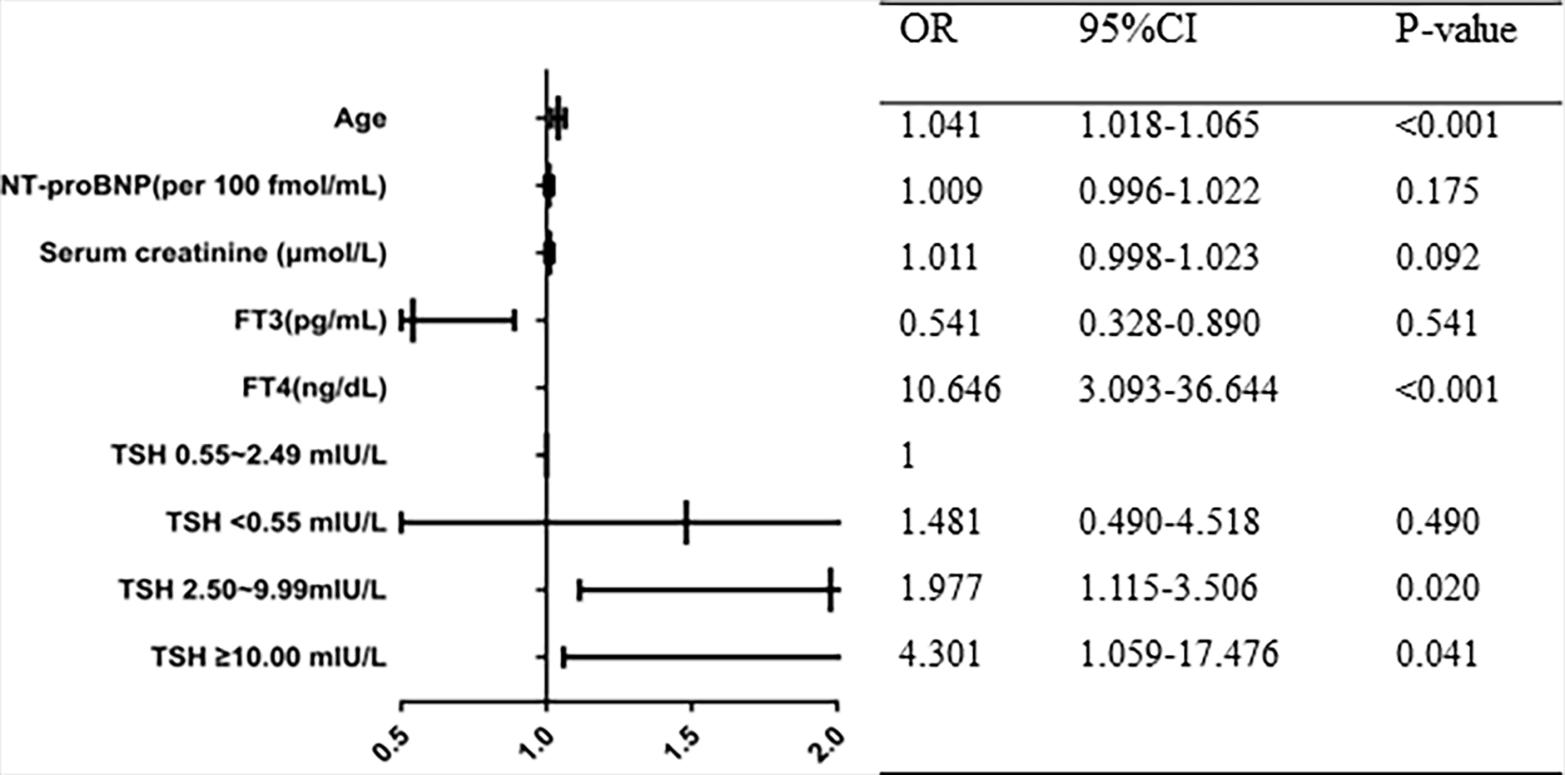
Multivariate logistic regression analysis of atrial fibrillation in patients with HOCM.

After adjusting for the common risk factor (age, NT-proBNP, serum creatinine, FT3, and FT4), stepwise multiple logistic regression analysis revealed that TSH levels were significantly related to AF incidence.Compared to patients with TSH (0.55 ~ 2.49 mIU/L) group, the adjusted odds ratio of AF for TSH(<0.55) group, TSH (2.50~9.99) group, TSH (≥10.00 mIU/L) group were 1.481 (95% CI 0.485~4.518,P=0.490), 1.977 (95%CI 1.115~3.506, p=0.02), 4.301 (95%CI 1.059~17.476, P=0.041), respectively (**Figure 1**).

## Discussion

This study explored the relationship between thyroid function and atrial fibrillation in patients with hypertrophic obstructive cardiomyopathy. In this study, the incidence of hypertrophic obstructive cardiomyopathy complicated with atrial fibrillation was about 14%, which was significantly higher than that of 2% - 4% in the general population (16), but lower than the previous hypertrophic cardiomyopathy in which the incidence rate of atrial fibrillation is 18-32% (17). Previous studies have shown that the prevalence and incidence rate of atrial fibrillation are different in different regions. The incidence rate of atrial fibrillation in the Asian population is lower than that in North America or Europe (18). This difference may be related to the underestimation of the prevalence of atrial fibrillation in the Asia Pacific region. At the same time, it also suggests that we should pay attention to the screening of atrial fibrillation in patients with hypertrophic cardiomyopathy.

This study found that the increase or decrease of TSH as a sensitive indicator of thyroid function can increase the occurrence of atrial fibrillation. In patients with hypertrophic obstructive cardiomyopathy complicated with abnormal TSH, the high incidence of atrial fibrillation may be due to the local or systemic effect of inflammatory mediators. At the same time, in univariate logistic regression analysis, it was found that the increase in age, the rise of NT-proBNP level, the rise in serum creatinine level, and the abnormality of FT3 and FT4 were significantly related to AF incidence. After adjusting for age, NT-proBNP, serum creatinine, FT3, FT4, and other risk factors, multivariate logistic regression analysis showed that the increase of TSH was an independent risk factor for atrial fibrillation in patients with hypertrophic obstructive cardiomyopathy and had predictive value for the prognosis of hypertrophic obstructive cardiomyopathy. This study further supports the hypothesis of thyroid hormone levels on hypertrophic cardiomyopathy.

In this study,the incidence of atrial fibrillation in TSH (0.55~2.49mlU/L) group was the lowest (10.20%), while TSH(> 10.00 mIU/L) group was the highest (35.29%). In TSH (< 0.55 mlU/L) group and TSH(2.50 ~ 9.99 mIU/L) group, the incidence of atrial fibrillation was 27.02% and 19.43%, respectively. It seemed that the incidence of atrial fibrillation in patients with hypothyroidism was higher than in patients with hyperthyroidism. The trend of my research results is similar to some previous studies. In a cohort study of 18021 patients with atrial fibrillation, 89% had normal thyroid function, 9% had hypothyroidism, and 2% had hyperthyroidism, suggesting that many patients with hypothyroidism also develop atrial fibrillation (19). Patients with hypothyroidism have abnormally high TSH levels and often insufficient T4 levels. Hypothyroidism also increases the risk of atrial fibrillation. With the deepening of research, researchers also realized that both hyperthyroidism and hypothyroidism will increase the risk of atrial fibrillation. This evidence involves thyroid hormone-induced changes in autoantibodies, inflammation, and ion channels. However, the mechanism of atrial fibrillation in patients with hyperthyroidism or hypothyroidism is very different. The arrhythmia of hyperthyroidism may be mainly due to the up-regulation of hyperdynamic circulation, cardiac structural and functional proteins, ion channels, and gap junction proteins (20–22). Hypothyroidism is associated with a variety of cardiovascular risk factors, such as metabolic syndrome, obesity, hypertensive heart disease, diabetes, and oxidative stress, which in turn can lead to atrial fibrillation (23–25). Hypothyroidism can reduce heart rate, prolong the atrial effective refractory period, increase atrial collagen in hypothyroid animals, and promote myocardial fibrosis. This leads to conduction heterogeneity and QT dispersion, which increases the risk of atrial fibrillation (26).

Some studies of non hypertrophic cardiomyopathy found that there is a certain relationship between TSH and the prevalence of atrial fibrillation. A previous study showed that (27), an apparent linear relationship between levels of thyroid dysfunction and atrial fibrillation risk—that is, a low atrial fibrillation risk in hypothyroid patients, a high risk in hyperthyroidism, and a TSH level-dependent (a dose-response relation) increased risk of atrial fibrillation in all levels of hyperthyroid disease, even in high normal euthyroid subjects. Notably, in subjects with reduced serum TSH levels but normal free thyroid hormone levels the risk of developing atrial fibrillation was increased by approximately 10% in individuals with high normal thyroid function and increased about 40% in those with subclinical hyperthyroidism with suppressed TSH levels. Another study shows that (28), the risk of AF increased with low normal TSH levels and slightly decreased with higher TSH levels (but remaining close to a hazard ratio [HR] of 1.0) compared to the reference level of 3.5mIU/l.

Previous studies have confirmed that different types of hypothyroidism, including subclinical hypothyroidism, low T3 syndrome, and clinical hypothyroidism, can affect the long-term prognosis of cardiovascular disease (29, 30). Animal experiments have confirmed that thyroid hormone has many effects on the cardiovascular system (31). Thyroid hormone can directly affect the metabolism and functional protein expression of cardiomyocytes and the remodeling of myocardial interstitium and microcirculation and electrophysiological disorders (32). In the state of hyperthyroidism, myocardial hypertrophy and a decrease of collagen fibers in the myocardial interstitium can be observed, which is related to the increase of matrix metalloproteinase-1 by thyroid hormone (33). Under the condition of hypothyroidism, collagen accumulation occurred in myocardial tissue. Thyroid hormone must affect the role of myocardial matrix collagen (34). In animal experiments, hypothyroidism has been shown to contribute to myocardial fibrosis and cause electrophysiological disorders (35). In 1992, Yao J et al. first reported that thyroid hormone could induce cardiac hypertrophy. This pathological change was characterized by reduced biosynthesis at type I collagen’s mRNA and protein levels (32). *In vitro* experiments conducted by Chen WJ et, al. showed that hypothyroidism could lead to an increase in the concentration of mRNA expressing pro-α1 (I) collagen, and this response can be inhibited by thyroid hormone receptors (TR-β1) (34). The conclusion of our study is consistent with that of the above basic research. It is well known that T3 is important for cardiac remodeling. In our study, for patients with obstructive hypertrophic cardiomyopathy complicated with atrial fibrillation, the FT4 value is high, but the FT3 value is low, and the possible mechanisms are diverse. Previous studies have shown that there may be obstacles in the process of T4 to T3 in the state of heart failure (36), We speculate that a similar mechanism may exist in patients with hypertrophic cardiomyopathy complicated with atrial fibrillation.

In this study, elevated TSH was an independent risk factor for atrial fibrillation in patients with HOCM. Therefore, the level of thyroid function should be regarded as an essential factor in evaluating the prognosis of HOCM. In addition, animal studies have shown that thyroid hormone replacement therapy can inhibit or even reverse cardiac cardiomyocyte fibrosis, which provides a further reference for the Future Treatment of hypertrophic cardiomyopathy, prevention of atrial fibrillation, and improvement of its prognosis (37, 38).

This study is a cross-sectional study; the sample size is limited, and there are some limitations. A large cohort study needs to verify further the correlation between thyroid hormone levels and survival and myocardial injury in patients with hypertrophic cardiomyopathy. However, this study found that abnormal TSH can predict the risk of atrial fibrillation in patients with hypertrophic obstructive cardiomyopathy, which can provide a reference for clinicians in the prognosis and treatment of patients with hypertrophic cardiomyopathy.

## Data Availability Statement

The raw data supporting the conclusions of this article will be made available by the authors, without undue reservation.

## Ethics Statement

The studies involving human participants were reviewed and approved by The Ethics Committee approved it of the Fuwai Hospital. The patients/participants provided their written informed consent to participate in this study.

## Author Contributions

Study concept and design: Y-DT, W-YW, XM, X-LW. Acquisition, analysis, or interpretation of data: Y-DT, XM, W-YW, KZ, JG, J-LZ, Z-YZ. Drafting of the manuscript: XM, X-LW, W-YW, KZ, CS, Y-DT, Z-YZ. Critical revision of the manuscript for important intellectual content: all authors. English language editing: KZ. Statistical analysis: XM, W-YW, X-LW, Y-PL, J-JW. Obtained funding: Y-DT. Study supervision: Y-DT.

## Funding

This work was supported by the National Key Research and Development Program of China (2020YFC2004700, 2020YFC2004705), National Natural Science Foundation of China (81800327, 81900272,81825003, 91957123), Beijing Municipal Commission of Science and Technology (Z181100006318005), and the Chinese Academy of Medical Sciences Innovation Fund for Medical Sciences (CIFMS 2016-I2M-1-009), Project of Henan Medical Science and Technology Research Program 2019 (LHGJ20190781), Beijing Municipal Commission of Science and Technology (Z171100000417021).

## Conflict of Interest

The authors declare that the research was conducted in the absence of any commercial or financial relationships that could be construed as a potential conflict of interest.

## Publisher’s Note

All claims expressed in this article are solely those of the authors and do not necessarily represent those of their affiliated organizations, or those of the publisher, the editors and the reviewers. Any product that may be evaluated in this article, or claim that may be made by its manufacturer, is not guaranteed or endorsed by the publisher.
